# Biochemical profile of paediatric adamantinomatous craniopharyngiomas: A prospective cohort study

**DOI:** 10.3389/fsurg.2022.1026926

**Published:** 2022-11-02

**Authors:** Luxwell Jokonya, Tariro Lavender Mduluza-Jokonya, Ignatius Esene, Garikai Mwale, Nqobile Sindiswa Thango, Takafira Mduluza, Thajasvarie Naicker

**Affiliations:** ^1^ Surgical Sciences Department, University of Zimbabwe, Harare, Zimbabwe; ^2^ Department of Optics and Imaging, University of KwaZulu-Natal, Durban, South Africa; ^3^ Department of Surgery, The University of Bamenda, Bambili, Cameroon; ^4^ Department of Neurosurgery, University of Cape Town, Cape Town, South Africa

**Keywords:** craniopharyngioma, craniopharyngioma cystic fluid, Africa Zimbabwe brain tumours, antimicrobials, sub-Saharan brain tumours

## Abstract

**Introduction:**

Craniopharyngioma is a rare brain tumour. Despite being histologically benign, it behaves aggressively and is often difficult to manage. Descriptive epidemiological data on the tumour is lacking in sub-Saharan Africa, and there is none for Zimbabwe. The tumour usually has a cystic component that has been raising interest in the past decade. Few studies have looked at the biochemical composition thereof. This study aims to give a landscape view of craniopharyngiomas (CPs) in Zimbabwe and then profile the biochemical properties of the cystic component of paediatric adamantinomatous craniopharyngioma.

**Methodology:**

A prospective cohort study was done in Zimbabwe over a 2-year period to study the epidemiological distribution of craniopharyngioma and examine the biochemical composition of adamantinomatous craniopharyngioma cystic fluid in the paediatric population. Fifteen patients were recruited who had craniopharyngiomas, and of those, nine paediatric adamantinomatous craniopharyngiomas had fluid analysed for biochemical components. SPSS statistical package was used to analyse the data. Descriptive statistics were used for epidemiological data.

**Results:**

The incidence of CP was calculated to be 0.53 per million person-years. Incidence among the paediatric population 0–14 years was 1.2 per 100,000 person-years. Several biological components were found to be elevated significantly compared to serum and cerebral spinal fluid (CSF). These are sodium, potassium, urea, alkaline phosphatase, phosphate, magnesium, albumin, gamma-glutamyl transferase, calcium, low-density lipids, and glucose.

**Conclusion:**

The incidence of CP in Zimbabwe is similar to the rest of the world. Some biochemical components have been noted to be markedly elevated in the cystic fluid and were mirroring serum rather than CSF in concentration.

## Introduction

Craniopharyngioma (CP) is a benign, brain tumour that commonly occurs in the suprasellar region of the brain. It has a worldwide incidence of 0.5–2 cases per million persons per year. This represents 1%–4% of tumours in the paediatric population. In the United States alone, around 338 cases occur per annum. There have been no ethnicity or gender differences noted. Epidemiological data for this tumour are difficult, not only because the tumour is rare but also because most cancer registries compute data on malignant tumours omit CP although it is borderline malignant. Furthermore, in the international classification for the disease of oncology, CP is classified under one code even though we now know that it represents dual pathologies. Adamantinomatous craniopharyngioma occurs predominantly in the paediatric population with a peak age of 5–14 years and is of embryonal aetiology (1–5).

In contrast, papillary craniopharyngioma occurs almost exclusively in the adult population with a peak incidence at 50–74 years and is thought to originate from metaplasia of remnants of Rathke’s pouch (6). Adamantinomatous CP exhibits a dysregulation of the Wnt signalling pathway while papillary is associated in 95% of cases with BRAF V600E mutations [valine (V) is substituted by glutamic acid (E) at amino acid 600]. These two are distinct, but because of the rarity of the tumour, most epidemiological studies tend to group them two together (7).

Of all the brain tumours, CP is perhaps the only one that has undergone radical changes in clinical management because of its associated profound challenges. Preferred management has shifted from radical to conservative resection, with an acceptance that this is a chronic disease, and the goal of therapy is to provide a good quality of life rather than a cure. The majority of options favour partial debulking of the tumour, intra-cystic therapies, and radiotherapy (8–13). Raised awareness of the cystic component for Ommaya reservoirs and intra-cystic drug therapies has resulted in several studies that explore the nature and contents of this cyst. We recently reported on the possible antimicrobial properties of the cystic craniopharyngioma fluid (14, 15). This study aimed to assess the biochemical components of the CP fluid to elucidate any antimicrobial properties.

## Methodology

### Setting and commencement

**Figure 1:**
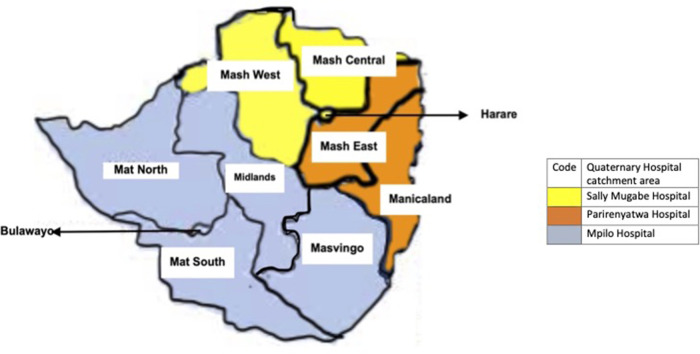
Map showing hospital catchment areas for neurosurgical patients in Zimbabwe.

A prospective cohort study was carried out to study the epidemiology of craniopharyngiomas and the biochemical composition of adamantinomatous craniopharyngioma cystic fluid over 2 years extending from July 2019 to August 2021. The catchment area for participants encompassed the entire country of Zimbabwe, a sub-Saharan country (Figure 1). Investigators had access to all government and private patients over the study period. At the time of the study, all neurosurgical government patients were being referred to the three quaternary hospitals as shown in Figure 1. All patients included in the study had a final diagnosis of craniopharyngioma. At the time of the study, the Zimbabwean population between the ages of 0–14 years was 42,000 and the total population was 14 million. The STROBE checklist was used in the write-up of the paper.

### Craniopharyngioma diagnosis

Diagnosis of craniopharyngioma was based on histological confirmation. In a few cases where biopsy for histology was unavailable, the diagnosis was made by a consensus of two of any combination of either a senior neurosurgeon and/or a senior radiologist based on typical radiological findings (including spectrometry), and if there was any doubt, then the diagnosis was not made. Otherwise, a histological result was the gold standard.

### Management of craniopharyngioma

The study did not influence or affect the standard management of craniopharyngioma which included resection, debulking, and Ommaya shunt insertion depending on the decision of the managing surgeon and the particular patient.

### Study inclusion criteria

All patients presenting to the three main teaching hospitals in the country (only state institutions offering neurosurgery services at the time of the study) with a diagnosis of craniopharyngioma were considered for study. Liaison with all neurosurgeons in the country (eight at that time) facilitate access to those patients from the private institutions (no CP patients were seen in the private sector during the period of study). Hence, all patients seen in the country with craniopharyngiomas could be captured.

### Ethical approval

Ethical approval was obtained from the Medical Research Council of Zimbabwe (MRCZ/A/1854). Informed consent was obtained from the participants or their guardians; assent was obtained for all children above the age of 7 years.

### Data collection

The patients (or caregivers) were interviewed. Past hospital documentation that included imaging and histology was reviewed. Demographic, history, examinations, investigations findings, and management information were captured. Patients with missing data would be excluded.

### Specimen collection and testing

#### Blood collection

Plasma and sera were obtained from blood collected in well-labelled ethylenediamine tetraacetic acid and coagulant-free blood collection tubes, respectively. Serum was tested for biochemical components.

#### CSF collection

CSF was collected during the insertion of a ventriculoperitoneal shunt when this was indicated for the treatment of the patient. After normal shunt insertion during the confirmation of the shunt working, some CSF fluid is allowed to drop out of the peritoneal end of the shunt.

#### Cystic fluid collection

Cystic fluid was collected, from the Ommaya reservoir, during scheduled drainage of the fluid or it was collected during surgical resection of the tumour.

### Specimen testing

The specimens were sent to a clinical pathology laboratory for biochemical analysis: urea and electrolytes, calcium, magnesium, phosphate, and liver function tests. All lipid measurements were performed in the same central laboratory. Serum concentrations of total cholesterol, high-density lipoprotein, low-density lipoprotein (LDL), cholesterol, and triglycerides were also measured.

The clinical management of the patients’ ailments was per hospital protocol, and the study did not interfere with their standard management.

### Statistical analysis

Data were then captured into a software package SPSS (SPSS, Chicago) for analysis. Descriptive statistics were used to summarise the patients’ demographical and clinical data. To determine statistical significance for parametrically distributed data, a *t*-test or one-way ANOVA with Bonferroni multiple comparison tests were used. For non-parametric data, a Mann–Whitney or Kruskal–Wallis test along with a Dunn’s multiple comparison test was used. Comparisons of proportions were performed using the Fisher exact and Chi-square tests. Significance was accepted with *p* values of <0.05. Data were expressed as median (90% confidence limits) as appropriate. Incidence was calculated using the formula: numberofonsets/totalperson−time.

## Results

### Demographics

**Figure 2:**
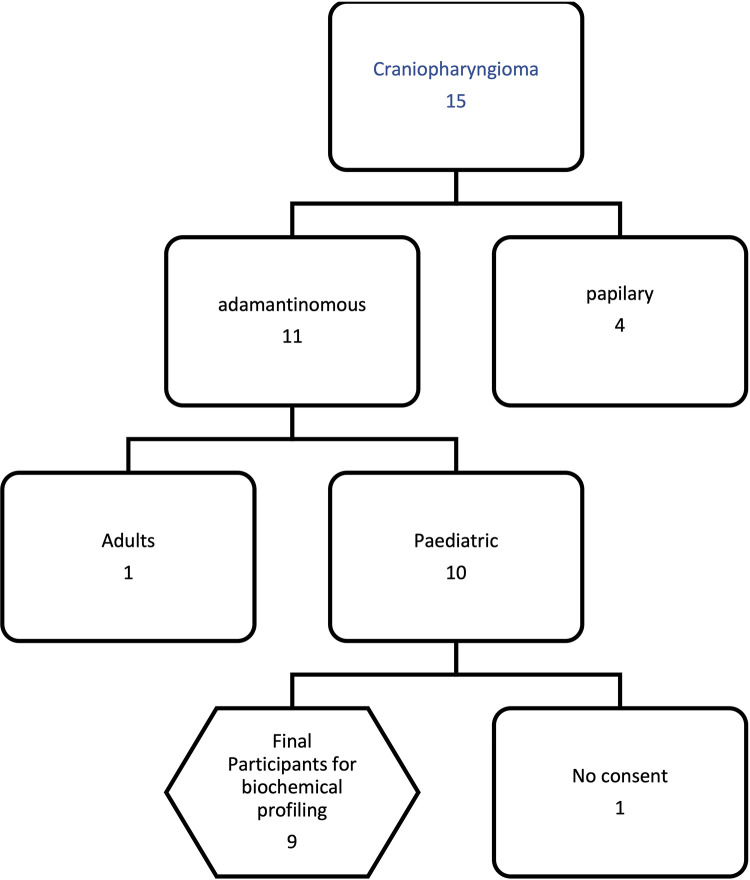
Study profile.

Of a total of 15 patients with craniopharyngioma, 9 patients were diagnosed with paediatric adamantinomatous craniopharyngioma and were recruited for fluid analysis in the study (Figure 2). The number of males was 6 (66.7%) and females were 3. The mean age is 9 ± SD was ±3.07 years and varied from 4 to 14 years (Table 1).

**Table 1:** Participant’s characteristics.

Characteristic	Total % (*n*)
Sex	
Males	66.7% (6)
Females	33.3% (3)
Age	
Mean (SD)	8.78 (3.07)
Sample origin	
Sally Mugabe Hospital	33% (3)
Parirenyatwa Hospital	11% (1)
Mpilo Hospital	55% (5)
Private Hospitals	0%

### Incidence of craniopharyngioma in Zimbabwe

Over the 2-year study period, a total of 15 patients were diagnosed. The Zimbabwe population was estimated at 14 million; hence, the incidence of craniopharyngioma was calculated to be 0.53 per million person-years. Incidence among the paediatric population 0–14 years was 1.2 per 100,000 person-years, which translates to five children in the 0–14 years age group are expected to present for the first time with craniopharyngioma annually in Zimbabwe.

### Craniopharyngioma cystic fluid composition

**Figure 3:**
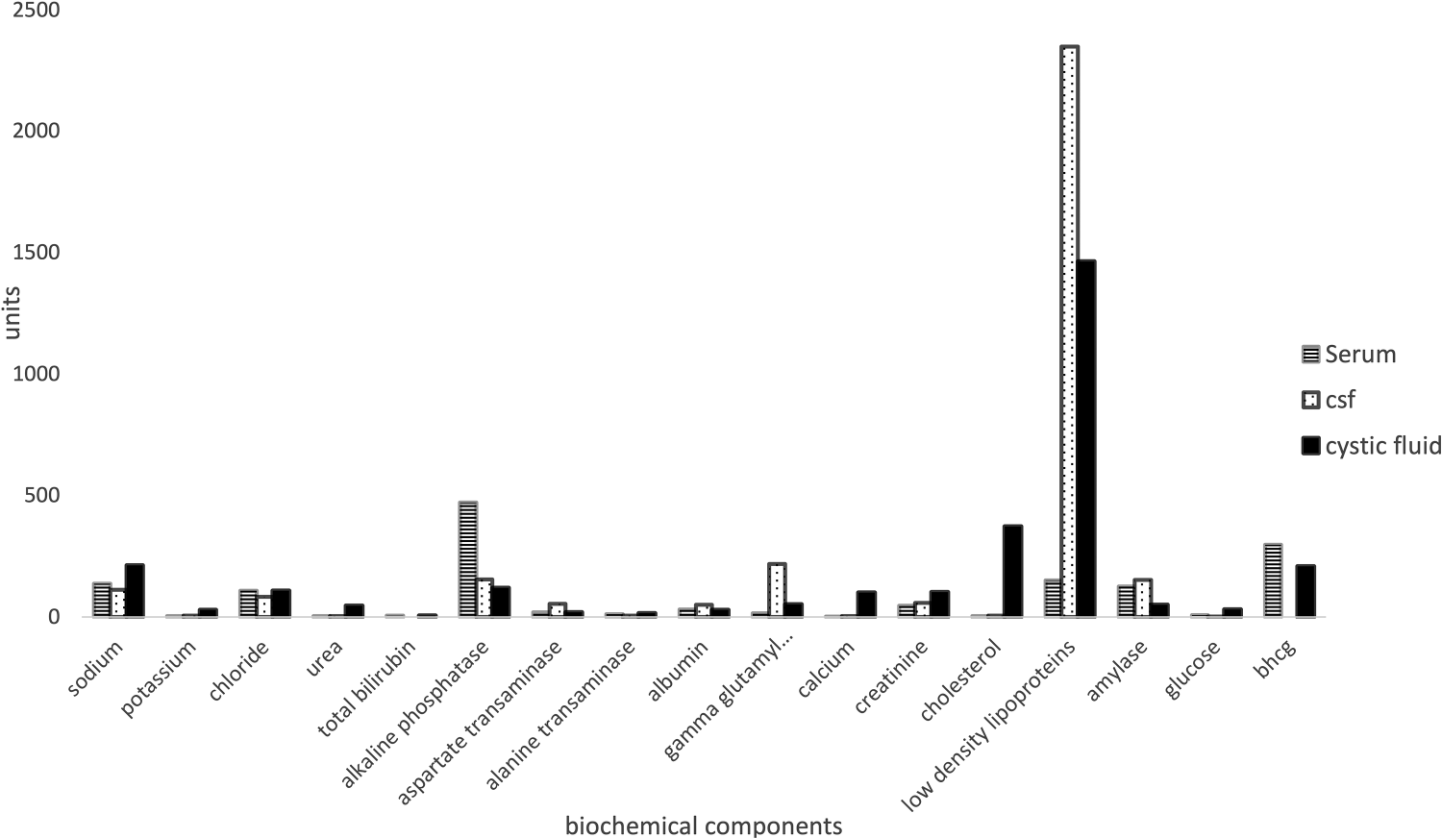
Relative concentrations between cystic fluid, serum, and CSF.

The following biochemical components were elevated in CP cystic fluid in comparison to the serum normal values: sodium, potassium, urea, alkaline phosphatase, phosphate, magnesium, albumin, gamma-glutamyl transferase (GGT), calcium, low-density lipoproteins, and glucose (Table 2). In addition, the cystic fluid also contained chloride, bilirubin, AST, ALT, creatinine, cholesterol, and amylase. The following biochemical components were present in all the fluids (serum, CSF, and the cystic fluid); sodium, potassium, chloride, urea, alkaline phosphatase, ALT, AST, albumin, GGT, calcium, creatinine cholesterol, LDL, amylase, and glucose (Table 3). Bilirubin and β-hCG were present in serum and the cystic fluid. The cystic fluid mirrored the serum as opposed to CSF (Figure 3, Table 2).

**Table 2:** Cystic fluid biochemical component means relative to normal serum ranges.

	Serum normal range	Mean cystic fluid levels (SD)
Sodium	135–145 mm/L	212 mmol/L (±18.1)
Potassium	3.6–5.6 mmol/L	28.3 mmol/L (±2.01)
Chloride	98–106 mmol/L	109 mmol/L (±17.13)
Urea	1.8–7.1 mmol/L	46.64 mmol/L (±13.72)
Total bilirubin	1.71–20.5 µmol/L	6.5 µmol/L (±1.3)
Alkaline phosphatase	32–92 IU/L	119 IU/L (±5.02)
Phosphate	2.7–4.7 mg/dL	14 mg/L (±1.3)
Aspartate transaminase	10–40 IU/L	18.78 IU/L (±2.3)
Alanine transaminase	5–40 IU/L	15.33 IU/L (±2.46)
Magnesium	1.74–2.4 IU/L	81 UI/L (±2.65)
Albumin	3.9–5.1 g/dL	28.2 g/dl (±3.56)
Gamma-glutamyl transferase	8.00–37 IU/L	51.44 IU/L (±8.4)
Calcium	2.7–4.5 mg/L	101 mg/dl (±1.3)
Creatinine	65.4–119.3 µmol/L	102 µmol/L (±2.0)
Cholesterol	>1.6 mg/L	373.11 mg/L (±31.52)
Low-density lipoproteins	140–280 IU/L	1463 IU/L (±37.1)
Amylase	30–110 IU/L	50.67 IU/L (±1.32)
Glucose	3.9–7.1 mmol/L	29.98 mmol/L (±11.3)

**Table 3:** Cystic biochemical components in relation to patient CSF and serum.

	Serum levels	CSF levels	Cystic fluid levels
Sodium	138 mmol/L (±7.11)	112 mmol/L (±18.1)	212 mmol/L (±18.1)
Potassium	4.09 mmol/L (±0.2)	5.9 mmol/L (±0.4)	28.3 mmol/L (±0.39)
Chloride	108 mmol/L (±3.35)	82 mmol/L (±1.81)	109 mmol/L (±7.13)
Urea	3.2 mmol/L (±0.3)	3.6 mmol/L (±0.65)	46.64 mmol/L (±13.72)
Total bilirubin	5.56 µmol/L (±0.13)	—	6.5 µmol/L (±1.3)
Alkaline phosphatase	471.78 U/L (±14.15)	154 IU/L (±9.12)	119 IU/L (±5.02)
Aspartate transaminase	19 IU/L (±2.8)	54 IU/L (±5.58)	18.78 IU/L (±2.3)
Alanine transaminase	12 IU/L (±2.52)	6 IU/L (±1.28)	15.33 IU/L (±2.12)
Albumin	31 g/dl (±5.86)	50 g/dl (±9.16)	28.2 g/dl (±4.4)
Gamma-glutamyl transferase	15 IU/L (±1.46)	219 IU/L (±13.91)	51.44 IU/L (±3.93)
Calcium	2.01 mmol/L (±0.32)	3.23 mmol/L (±0.13)	101 mg/dl (±3.2)
Creatinine	46 µmol/L (±3.29)	57.8 µmol/L (±10.46)	102 µmol/L (±7.31)
Cholesterol	2.9 mmol/L (±0.17)	4.9 mmol/L (±0.91)	373.11 mg/L (±8.45)
Low-density lipoproteins	150 IU/L (±11.37)	2349 IU/L (±9.86)	1463 IU/L (±37.11)
Amylase	125 IU/L (±6.88)	153 IU/L (±13.5)	50.67 IU/L (±1.32)
Glucose	7.43 mmol/L (±1.01)	0.3 mmol/L (±0.06)	29.98 mmol/L (±10.09)
β-hCG	297 mIU/ml (±13.72)	—	209 mIU/ml (±11.51)

## Discussion

This paper presents for the first time the incidence of craniopharyngiomas in the paediatric population in a sub-Saharan country, Zimbabwe. It was calculated to be 0.53 per million person-years and in accordance with global estimates. However, the incidence among the 0–14-year age group is twofold higher than in other countries, with five children expected to present with CP every year. This is very worrisome given that Zimbabwe is a low-income country with relatively low health services. It is plausible that this elevation may be that the actual incidence of CP tumours in sub-Saharan Africa is high and also have high mortality (preventing them from getting into adulthood). A follow-up study to determine 5- and 10-year survival of these patients is urgently required to confirm these data.

Our study showed a male preponderance of 2:1. While American and Finland data showed no gender preference, a large hospital-based study in the United Kingdom reported a male preponderance with a ratio of 1.3–1.6 in the paediatric population (1, 11). We noted a slightly higher number of cases from the southern part of the country (Mpilo hospital) albeit this difference was statistically non-significant.

We report that CP cystic fluid had elements found in both CSF and serum, though the quantity was significantly elevated in the cystic fluid. This finding is significant as it is evident that the fluid is actively secreted by tumour components as opposed to past studies which suggested that the fluid was transudate. We report elevated urea and electrolytes compared to the serum values of the same participants. The level of sodium was reported to be twice as high as in the serum of CP patients. Sodium is reported to have antimicrobial properties, particularly in lactic acid-producing bacteria such as *Staphylococcus aureus* and *Salmonella* (16–20). Additionally, potassium was elevated fourfold that of serum levels. Moreover, chlorine a halogen that is widely used for inactivating microorganisms was similar in CP cystic fluid compared to that in serum. Also, urea concentration in the CP cystic fluid was found to be elevated 15-fold than that of serum levels. Creatinine was twofold elevated in CP cystic fluid in comparison to its value in the serum of CP patients. Calcium, phosphorus, and magnesium account for 98% of the body’s mineral content by weight. We report that magnesium was 40 times elevated in CP fluid compared to the serum. Phosphate was threefold higher in CP fluid compared to serum. This study aimed to elucidate the biochemical components of adamantinomatous CP cystic fluid with the view of possible antimicrobial components as a follow-up to a previous study that had shown significant antimicrobial activity in the fluid (11–13). All these biochemical components have different antimicrobial properties among other things (21–31*).*

The CP cystic fluid is also termed machinery oil fluid particularly because of its elevated oil levels. Low-density lipoproteins (LDL) were 100 times elevated in CP cystic fluid compared to serum. In our study, cholesterol was 100 times elevated in the CP compared to serum. This finding can be potentially useful in spectrometry to diagnose CP in the absence of histology (34, 35). In the same vein, β-hCG can potentially be used to aid in diagnosis as well. In addition, glucose was four times elevated in CP cystic fluid, in contrast to amylase which is lower in CP fluid than serum. Biochemical profiling of the cystic fluid composition can potentially assist in the development of targeted intra-cystic therapeutics.

To the best of our knowledge, amylase is being reported for the first time in craniopharyngioma cystic fluid in this study. Studies on the biochemical composition of the cystic fluid are scanty, and there have been reports of lipids, proteins, glucose, urea, creatinine, sodium, calcium, magnesium, phosphorous, chlorine, ALT, AST, and GGT, but none on amylase (7, 8, 14, 34, 35). The significance of this important finding remains to be elucidated.

The study strength is that the catchment area encompassed the entire country and we managed to confirm most of the diagnoses histologically. Some of the limitations of the study are that some patients, particularly those who seek help in traditional medicine or foreign countries, could have been missed. More specimens would increase the strength of the study; however, craniopharyngioma is classified as a rare disease, hence the small participant size (1, 2).

## Conclusion

Our study demonstrates for the first time the prevalence of CP in a sub-Saharan country, Zimbabwe, to be 0.53 per million person-years. This study demonstrates higher concentrations of sodium, potassium, urea, bilirubin, creatinine, cholesterol, and low-density lipoproteins in CP fluid compared to serum; this better understanding of the cystic fluid composition has a bearing on the further development of targeted diagnostics and therapeutics in this difficult to manage tumour.

## Data Availability

The raw data supporting the conclusions of this article will be made available by the authors, without undue reservation.
